# Applications of Model-Based Meta-Analysis in Drug Development

**DOI:** 10.1007/s11095-022-03201-5

**Published:** 2022-02-16

**Authors:** Phyllis Chan, Kirill Peskov, Xuyang Song

**Affiliations:** 1 Clinical Pharmacology, Genentech, 1 DNA Way, South San Francisco, CA 94080 USA; 2 M&S Decisions LLC, Moscow, Russia; 3 Sechenov First Moscow State Medical University, Moscow, Russia; 4 STU ‘Sirius’, Sochi, Russia; 5 Clinical Pharmacology and Quantitative Pharmacology, AstraZeneca, 1 Medimmune Way, Gaithersburg, MD 20878 USA

**Keywords:** Meta-analysis, Competitive benchmarking, Model-informed drug development, Durvalumab, Fenebrutinib

## Abstract

Model-based meta-analysis (MBMA) is a quantitative approach that leverages published summary data along with internal data and can be applied to inform key drug development decisions, including the benefit-risk assessment of a treatment under investigation. These risk–benefit assessments may involve determining an optimal dose compared against historic external comparators of a particular disease indication. MBMA can provide a flexible framework for interpreting aggregated data from historic reference studies and therefore should be a standard tool for the model-informed drug development (MIDD) framework.

In addition to pairwise and network meta-analyses, MBMA provides further contributions in the quantitative approaches with its ability to incorporate longitudinal data and the pharmacologic concept of dose–response relationship, as well as to combine individual- and summary-level data and routinely incorporate covariates in the analysis.

A common application of MBMA is the selection of optimal dose and dosing regimen of the internal investigational molecule to evaluate external benchmarking and to support comparator selection. Two case studies provided examples in applications of MBMA in biologics (durvalumab + tremelimumab for safety) and small molecule (fenebrutinib for efficacy) to support drug development decision-making in two different but well-studied disease areas, i.e., oncology and rheumatoid arthritis, respectively.

Important to the future directions of MBMA include additional recognition and engagement from drug development stakeholders for the MBMA approach, stronger collaboration between pharmacometrics and statistics, expanded data access, and the use of machine learning for database building. Timely, cost-effective, and successful application of MBMA should be part of providing an integrated view of MIDD.

## Introduction

During clinical drug development, the primary efficacy and safety outcomes of clinical trials conducted by the sponsor typically determine the selected dose of a novel molecule, as individual patient data provide a rich source of information. However, benefit-risk assessments of the treatment under investigation are often made with limited internal data. Using internal data along with external data may improve internal decision-making and decrease the failure rate of the clinical trials for novel therapeutics. Model-based meta-analysis (MBMA) is a quantitative approach that leverages external published summary data for indirect treatment comparisons and may be used to determine an appropriate dose relative to established comparators. Hence, MBMA is a critical component of a successful model-informed drug development (MIDD) framework.

**Fig. 1:**
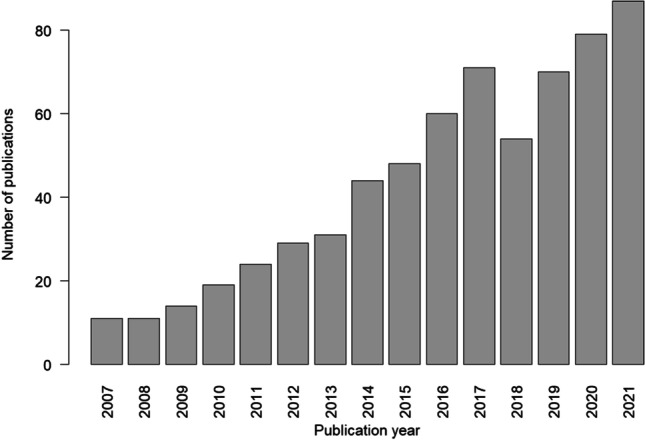
Bar chart of PubMed.gov search results of MBMA by year

Despite the essential value that MBMA could provide to the drug development process, the role, and contributions of MBMA within MIDD have not been recognized as much as conventional statistical meta-analysis as shown in the PubMed search on "model-based meta-analysis" and “meta-analysis” (620 vs. 226,627 citations respectively, as of October 22, 2021). However, Fig. 1 indicates an increase in the number of MBMA papers in PubMed.gov, which reflects recent interests in the field. Additional recognition and engagement from drug development stakeholders for the MBMA approach are essential to continue progress in the timely, cost-effective, and successful applications of MBMA.

One of the challenges that contribute to the scarcity of MBMA publications and applications is that decision makers in the drug development process may not be sufficiently trained to interpret MBMA results. At times, a lack of understanding of the assumptions and uncertainties in the MBMA approach may translate to general skepticism regarding the value of MBMA. A "communication gap" that exists across disciplines, both within industry and between industry, and regulators regarding MIDD approaches appears to be another reason for the relatively scarce application of MBMA. In a survey conducted in 2017 among clinical pharmacology and pharmacometric colleagues across the industry, United States Food and Drug Administration (US FDA), and European Medicines Agency (EMA) to understand current and future roles of MIDD, the authors of the study suggested that key stakeholders in industry and regulatory authorities become more engaged in MBMA training in order to gain additional trust and confidence this type of quantitative evaluation to provide indirect comparative efficacy and safety information [1].

Publications about the use of MBMA in a regulatory context have been limited. The US FDA published a draft guidance for conducting meta-analysis to evaluate the safety of human drugs or biological products [2], but currently there is no specific guidance on MBMA. Similarly, the sixth iteration of the Prescription Drug User Fee Act (PDUFA VI), which was reauthorized in 2017 as part of the US FDA Reauthorization Act (FDARA) through September 2022, includes the evaluation of model-based strategies to support drug development but contains only a single sentence on meta-analysis, without specific mention of MBMA [3]. In addition, the Efficacy Working Party of the EMA published a Points-to-Consider guideline, which discusses the difficulty in the validity and interpretation of the meta-analysis in applications for the marketing authorization of medicinal products and use of meta-analysis or MBMA for drug development decisions was out of the scope of the guideline [4]. Nevertheless, a more prominent endorsement and promotion of the benefits of MBMA from global regulatory agencies could greatly boost application.

Here we provide an overview of meta-analysis concepts, focusing on the various aspects of model-based meta-analysis, as well as two case studies to demonstrate the successful applications of model-based meta-analysis in clinical drug development.

## Types of meta-analyses

### Pairwise Meta-Analysis

Although analyzing data from individual clinical trials remains the gold standard for evaluating the efficacy and safety characteristics of a drug, meta-analysis of aggregated published trial data is a commonly accepted statistical technique in drug development to supplement individual-patient level analyses. Meta-analysis is routinely leveraged to provide a more precise estimate of the overall treatment effect compared to the individual clinical trials contributing to the pooled analysis, to evaluate efficacy and safety response in a subgroup of patients and to improve the estimation of the dose–response relationship [4].

Definitions and techniques of meta-analysis have evolved with increasing specificity and complexity. Standard pairwise meta-analysis (PMA) began to appear regularly in the medical literature in the late 1970’s and is recognized as the highest level in the hierarchy of evidence for evidence-based medicine, as the approach involves a strictly defined pool of similar studies from a systematic review [5].

PMA is limited to comparisons of two treatments at a time, using treatment arms directly evaluated in head-to-head trials. Variability in efficacy or safety response is expected among different clinical trials comparing the same treatments, and a weighting scheme is implemented to reflect the different values of evidence source [5]. However, in order to reduce the heterogeneity in the data, studies included in a PMA tend to have similar patient populations in terms of the entry criteria for the studies [6].

### Network Meta-Analysis

Network meta-analysis (NMA) extends the principles of PMA to include the evaluation of more than two treatments simultaneously. In addition to direct comparisons based on data from randomized controlled trials, indirect comparisons of treatments that were not investigated in the same clinical trial can be inferred through a common comparator treatment, such as placebo or standard-of-care (SOC)[7, 8]. Because of this ability to combine direct and indirect comparisons, NMA is useful as a competitive benchmarking tool to examine the comparative effectiveness of multiple treatments, including comparing an investigational drug to multiple previously approved SOC for a well-researched disease area, or when comparing multiple experimental molecules that are still under development for the same indication [7]. The application of NMA is far-reaching in drug development; NMA is widely used within the pharmacoeconomics community of practice [9] and is increasingly being used by reimbursement agencies to inform decisions about relative efficacy and cost-effectiveness based on late stage and post-marketing evidence [10].

When conducting NMA, a network graph is constructed to provide an overview of the amount of information available for each treatment including the following: the most common comparator treatment, the number of studies comparing each pair of treatment, the number of studies and the number of subjects included in the treatment [11]. Information between different drugs from the same class or with the same mechanism of action (MOA) can be leveraged, if deemed valid, but the inferences made using NMA require additional assumptions and data than for conventional PMA. One major limitation of NMA is that it does not incorporate longitudinal data. Therefore, studies vary substantially in treatment duration cannot be sensibly incorporated into a NMA [12].

### Model-Based Meta-Analysis

Meta-analyses that include statistical models for longitudinal disease data or pharmacologic concepts, such as dose–response relationships, are often referred to as MBMA. Establishing a dose- or exposure–response (E-R) relationship is critical to inform early go/no-go development decisions. Because an MBMA is able to integrate more comprehensive information from relevant, publicly available, summary-level efficacy and safety data from external clinical trials to augment internal data, it can potentially be applied earlier in the drug development process to help forecast future outcomes and thus provide a more timely benchmarking relative to simpler PMA or NMA based on mature trial readouts[13].

A common application of MBMA is the selection of optimal dose and dosing regimen of the internal investigational molecule to evaluate external benchmarking and to support comparator selection [9, 13]. The relative dose–response relationships established by the MBMA can be compared by using an overall effect describing the sum of the drug effect, placebo effect, and model parameters describing the shape of the dose–response curve. The results of MBMA should be particularly checked for the differences between the observed change from baselines and the predicted change from baseline to ensure there is no systematic under- or over-prediction in respect to drug class, drug, study, or duration [11].

As in PMA and NMA, statistical rigor that exists in meta-analysis in general should be followed in MBMA, especially regarding disciplined literature search and model evaluation as outlined in the Cochrane handbook, in order to provide a consistent approach to the conduct of systematic review [14].

#### Longitudinal Data

In a basic PMA or NMA, only data at the primary study endpoint is used; intermediate time points are excluded from the analysis. However, incorporating longitudinal information allows evaluation of the full time-course of the response in terms of both its rate of onset and its magnitude, and this feature of MBMA could provide more accurate estimates of the true response and thereby a more valid comparison between treatments [15]. MBMA leverages principles from the nonlinear mixed effects modeling approach to handle multiple and correlated observations collected from each trial arm in longitudinal data [16, 17]. Time-course of response is often fitted using an Emax model, potentially including the maximal effect (Emax), steepness of the curve (Hill coefficient), and the time associated with 50% of maximal effect (ET50) for individual treatments, if sufficient data is available [10]. To describe the dose–response relationship, an Emax model, a simpler linear, exponential, or even stepwise (on/off) model can be used, especially with external summary-level data, where information from multiple doses might be scarce. Based on the assumption that treatments with the same MOA should exhibit similar level of maximal effect after target saturation, a common time-course effect model is often used to fit the longitudinal data from all treatments of the same class.

In addition to modeling the dose–response relative to placebo or SOC, the longitudinal data of placebo can also be evaluated using MBMA, independent of active treatment data, to describe natural disease progression using summary-level data. Subsequently, the disease progression models based on MBMA could be supplemented with the time-course of treatment effects, and the combined model can be leveraged for clinical trial design decision purpose by identifying the shortest duration of a trial that would enable adequate resolution of treatment effect [9].

#### Combining Individual- and Summary-Level Data

As aggregated individual-level data is the gold standard for characterizing the efficacy or safety response, a potential rich source of individual-level data for building a disease progression model comes from disease research groups or consortia, which are based on the collaboration of multiple research entities. However, often the consortia database only contains data in the placebo or SOC arms but not in the investigational arm of clinical trials, and relevant patient-level data in the active treatment arms from external sources are difficult to obtain for many sponsors [5]. Therefore, an opportunity exists to combine individual-level data, either from a disease progression database or from internal clinical trial data, with the summary-level data from literature sources in a MBMA. This approach would allow a comprehensive analysis of available evidence, while also enabling the simulation of realistic patient-level data in a clinical trial [18].

Although combining individual- and summary-level data increases the data resolution for a more thorough data analysis and MBMA model building, one drawback is the complexity of integrating statistical assumptions across data types and potential to introduce bias, as summary-level data for a meta-analysis is collected with specific study aims and may or may not be representative of the individuals that make up a disease population [19]. If the correlation between time points for individual patients are assumed to be the same as those for treatment arms, this might lead to “ecological bias” as there is no guarantee that correlations at the individual level would be the same as at the aggregate level [20]. For example, when using summary-level efficacy data to construct a MBMA model in Alzheimer’s Disease, the time-course of expected disease progression of the Alzheimer's disease assessment scale cognitive subscale (ADAS-cog) scores for "average individuals" showed negligible nonlinearity, even though nonlinearities are apparent at the individual level [18]. Therefore, when combining individual- and summary-level data in a MBMA, additional estimations and assumptions are required to differentiate study-level and patient-level variability terms, so that the equation for the likelihood describing the individual-level data is structured differently than the one describing the probability in the summary-level data [19].

Due to the consideration for model assumptions and increased complexity in model development, based on the authors’ experience, the implementation combined individual- and summary-level data for a MBMA is not common in practice. In order to reduce model assumptions and expedite the completion of the analysis to support end-of-phase II go/no-go decisions, typically internal patient-level data are also summarized to the study arm level to be aggregated with external data for MBMA.

#### Building a model-based meta-analysis database

A critical step of any meta-analysis is to ensure that the literature search is comprehensive and the analysis database is relevant to the objectives, and a database building protocol should be pre-specified. Database searches should include sources from relevant scientific journals, books, ClinicalTrials.gov entries, and conference abstracts and posters. When exploring clinical trial results, recognition of potential biases—such as poor quality of study design (unblinding or randomization), lack of publication from studies with negative outcomes, and patients in earlier clinical trials are more likely to have received a different type of SOC and non-pharmacological treatments—are necessary in deciding if certain source data should be included for a meta-analysis. Such biases are likely impact the estimation of treatment effects. An appreciation and understanding of the competitive landscape and the endpoints of interest and cross-functional input from treatment landscape subject matter experts is essential for successful application of MBMA.

In addition to the publication title, type, and year, a MBMA database should include treatment name, dose, frequency, and duration at the minimum [21]. Efficacy and/or safety data based on the endpoints of interest would typically be pre-specified, after discussions with the project team, in particular the clinical science and epidemiology functions. Depending on the objectives of the analysis, summary-level covariate information, such as the proportion of a categorical variable (e.g. gender, prior treatment status) or the average of a continuous measurement (e.g. age, body weight), would also be extracted from the publications.

In addition to extracting data reported in literature, database augmentation and methods to standardize variables, such as variability terms and treatment dose amount across citations, variable naming conventions, and to impute missing values are expected. Imputations can be performed by deriving standard error based on published standard deviation and sample size information, or for missing covariate data by leveraging available information from similar patient demographics in other trials. Another consideration for database building that is specific to MBMA is digitizing longitudinal data, which can introduce additional error [17]. A documentation of the literature search process for constructing the MBMA database and of the inclusion/exclusion rationale for building the subsequent analysis dataset is required in order to justify decisions such as the endpoint chosen for the analysis, which can depend on data availability, and the exclusion of studies with very small sample size (e.g. N = 20), as the patient population in larger clinical trials are more heterogeneous and potentially more informative for covariate analysis.

A rigorous methodology using core standards developed by the Cochrane Collaboration should be followed, including establishing a clear protocols for literature search and diagnostic methods for evaluating the analysis outputs [22]. A flow diagram is often useful for capturing the number and reasons of exclusions to arrive at the studies extracted for the MBMA database and data included in the analysis dataset [23]. If the application of the MBMA is intended for competitive benchmarking that includes an investigational treatment with an upcoming clinical trial readout, much of the database building and even the model building efforts can be accomplished in the preparatory stage, while waiting for the data from the particular clinical trial to become available. In this manner, the MBMA could be re-run quickly with the data from the investigational treatment of interest and the model-based comparative efficacy results would be available to support drug development decisions in a timely manner.

#### Data resolution for covariate analysis

*C*ommon applications of MBMA include supporting selection of patient subpopulations for a clinical trial design. This is feasible because, in contrast to PMA, which tends to select studies with similar patient populations in order to reduce the heterogeneity in the data, one of the major benefits of MBMA is that the impact of covariates on the treatment effect is routinely incorporated into the model [24]. Therefore, the differences in design and population characteristics that could lead to heterogeneity in the treatment effects between studies are desirable in some respects for a MBMA. However, the few numbers of covariates reported and the low resolution of the covariates in literature data may restrict the depth of covariate investigation.

To reduce the likelihood of spurious patient factor associations, a list of clinically plausible covariates should be pre-specified prior to conducting covariate analysis in MBMA model development, and random exploration of covariate effects should be avoided. Particularly, in a small MBMA database with data from fewer trials, there might not be enough power to detect the covariate effects, resulting in a greater risk for spurious findings [24].

Across various published MBMA in literature, almost all the analyses were performed using NONMEM, R, and/or BUGS (Bayesian inference Using Gibbs Sampling) software. Evaluation of the analysis results includes a forest plot showing the predicted effect relative to placebo from each study, with the observed estimate and 95% confidence interval (CI). Any major differences between the observed values and the predicted estimates in the forest plot are scrutinized to explain potential sources of the deviation, such as study conduct modifications or non-standard study outcome reporting. This type of plot can help identify covariates that make certain study arms different other arms.

## Case studies

The following two case studies will be used to demonstrate the different applications of MBMA in supporting various aspects of drug development in two well-studied disease areas.

### Case study 1: Comparative MBMA of ICI safety data in monotherapy and combination setting

Multiple clinical trials of immune checkpoint inhibitors (ICI) such as cytotoxic T lymphocyte–associated antigen 4 (CTLA-4) inhibitors, programmed cell death protein 1 (PD-1) inhibitors, and programmed death-ligand 1 (PD-L1) inhibitors, and their combinations published in the last decade created a unique landscape for the application of the MBMA techniques [25]. The value of extracting clinically useful safety information via meta-analysis from oncology clinical trials has been previously reported in a detailed and systematic manner [26].

Adverse event (AE) incidence has been comprehensively evaluated for various organ classes including skin, gastrointestinal, renal, hepatic, pulmonary, and others [27]. Additionally, it has been shown that the safety profiles of immune-mediated AEs (imAEs) differ for the anti CTLA-4 and anti PD-L1 monotherapies and the AE incidence is further increased under combination of these agents [28, 29]. Despite such a substantial amount of clinical ICI safety data, the exact imAEs mechanisms underlying pathophysiology and quantitative relationship with ICIs exposure are still not fully understood [30]. Therefore, the primary objective of the MBMA was to establish a quantitative framework for the analysis of ICI exposure and dosing regimen effect on the incidence of specific AE rates.

#### Exposure-safety meta-analysis of immune checkpoint inhibitors

Following the Preferred Reporting Items for Systematic Reviews and Meta-Analyses (PRISMA) guidelines, a total of 102 eligible articles and abstracts data sources published from 2005 to 2018 were selected for preparation of the modeling dataset [31]. The selected articles included 153 treatment cohorts of 21,305 patients who received PD-1 or CTLA-4 ICI monotherapy or combination therapy across 80 clinical trials. Publication bias analysis via Funnel Plots and respective Egger’s statistical test [32] revealed no significant heterogeneous asymmetry, indicating no obvious publication bias with respect to both total treatment-related AEs (trAE) and tissue-specific imAEs.

**Fig. 2:**
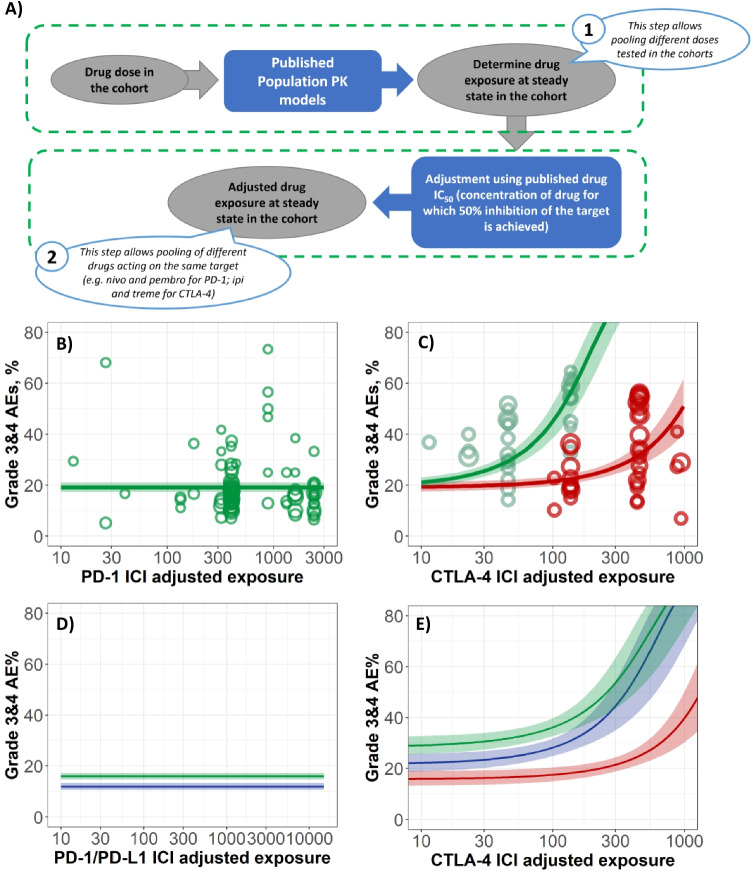
Case study 1: Comparative MBMA of ICI safety data in monotherapy and combination setting. **A** Estimation of adjusted ICIs exposure: step 1, using published population PK models to simulate typical ICI exposure in the cohort; step 2, exposure adjustment based on the published ICIs potency data. **B** Exposure-safety dependence of total grade 3/4 AEs upon PD-1 monotherapy. **C** Exposure-safety dependence of total grade 3/4 AEs upon CTLA-4 mono- (red) and CTLA-4 + PD-1 combination (green) therapy. Individual trials used for model calibration are shown with circles, diameter corresponds to sample size, and 90% confidence interval are represented by respective bands. D) Simulation of exposure-safety dependence of total grade 3/4 AEs upon PD-1 (green) and PD-L1 (blue) monotherapy. E) Simulation of exposure-safety dependence of total grade 3/4 AEs upon CTLA-4 mono- (red), CTLA-4 + PD-1 (green) and CTLA-4 + PD-L1 (blue) combination therapies

A meta-analysis was performed based on a random-effects meta-regression approach with logit-transformed AE rates. For the AE types whose averaged incidences were less than 5%, a normal-binomial general linear mixed model was used, as normal distribution assumption for within-trial variability is not valid for rare events [33]. In order to combine AEs across different ICI dosing schedules, a new model-based approach was proposed to account for the differences in ICI exposure and target receptor binding, which can address dosing schedule differences among comparators (Fig. 2A). In particular, published population pharmacokinetic models were used to simulate trough drug concentration at steady state to obtain a typical ICI exposure from each study cohort. The simulated exposure was then adjusted by the drug concentration in vitro measured IC50 values, which were also taken from the published source. Adjustment of drug exposure by the drug IC50 value allowed the aggregation of AE data from different ICIs binding on the same target receptor (Fig. 2A). In order to assess the key factors influencing the between trial variability the base model incorporating dose/exposure dependence was further followed with sequential (forward and backward) step-wise covariate search [31].

In accordance with the results of single trials, the model analysis revealed no significant dose/exposure dependence for AEs of PD-1 inhibitor monotherapy [34] (Fig. 2B). Conversely, CTLA-4 inhibitor dose/exposure led to significant increases in AEs incidence. Interestingly, that for PD-1 inhibitor + CTLA-4 inhibitor combination therapy, AE rates increased intensively and with less dose/exposure of CTLA-targeting agent (Fig. 2C). Thus, the structural model that best described the total grade 3/4 trAE rate dependence on adjusted ICI exposure was the model accounting for the synergistic component, where the total grade 3/4 trAE rate was driven by CTLA-4 inhibitor exposure with a PD-1 inhibitor dependent modulation. This model element facilitated the explanation of the significantly higher AE rate observed with the combination treatment, despite the lower CTLA-4 inhibitor typical exposure in combination therapy versus monotherapy.

Meta-regression analysis was also used to determine covariates influencing ICI safety profile and its dose/exposure dependence. Thus, line of treatment and combination with chemotherapy were found to be statistically significant. This allowed to prospectively predict total grade 3/4 trAE rates for treatment options that have not yet been tested in clinical trials at time of analysis. In particular, a specific treatment of interest consisting of a triple combination of PD-1 inhibitor + CTLA-4 inhibitor + standard chemotherapy, which was predicted to achieve a grade 3/4 trAE rate of 50% at lower doses than doses tested in clinical trials of ICI dual combination therapy [31]. Interestingly, there were no statistically significant differences in AE rates across different cancer types. This finding suggests that ICI-related AE incidence across cancer types may not significantly differ and supports the practice of pooling ICI AE data across different cancer types when performing MBMA [31].

#### Comparative safety analysis of durvalumab + tremelimumab combination

At the second stage of the analysis, the MBMA database was extended using data from studies with PD-L1 inhibitors given as monotherapy or in combination and from additional clinical studies with other ICIs published from 2018 to 2020. This led to the compilation of a dataset containing a total of 201 clinical studies with 400 cohorts and 64,471 patients, which to our knowledge, is the largest dataset to date, constructed based on publicly available clinical aggregate data [35].

The implementation of the previously mentioned novel methodology on the database extension led to results consistent with those from the first analysis. Specifically, no exposure dependence on safety was found in PD-1 or PD-L1 inhibitors given as monotherapy, and combination therapy of PD-1 + CTLA-4 inhibitors or PD-L1 + CTLA-4 inhibitors is associated with higher AEs frequency than that of CTLA-4 monotherapy. Interestingly, the frequency of grade 3/4 trAEs with PD-L1 inhibitors given as monotherapy was lower compared with that of PD-1 inhibitors given as monotherapy, 12.4% and 16.1%, respectively (Fig. 2D). In the combination therapy setting, CTLA-4 inhibitors combined with anti PD-L1 agents also showed lower trAEs frequencies than combinations including PD-1 blocking ICIs (Fig. 2E). This observation is in the good agreement with prior reports and can be caused by the ability of PD-1 antagonists to block binding with both ligands, PD-L1 and PD-L2, while PD-L1 agents unleash only PD-1/PD-L1-driven peripheral immune tolerance [36, 37]. These MBMA results were used to support the phase III dose justification of an upcoming combination therapy study of durvalumab + tremelimumab.

### Case study 2: Clinical trial design using an early efficacy endpoint and competitive benchmarking of fenebrutinib in patients with rheumatoid arthritis

Rheumatoid arthritis (RA) is a well-studied disease area with a large number of therapies across various MOAs showing efficacy, thus providing an opportunity to conduct MBMA based on the abundance of publicly available data as a part of a whole suite of MIDD analyses to address different clinical trial questions at various stages of the clinical drug development.

#### Selection of an early efficacy endpoint in a rheumatoid arthritis clinical trial

Generally, for registrational trials in RA, the efficacy result after 6 months of treatment is regarded as the primary endpoint, whereas an earlier endpoint based on short-term data may be preferred in early-stage proof-of-concept (PoC) trials to enable earlier go/no-go decisions. In order to assess the feasibility of predicting 6-month efficacy from short-term data, an MBMA was performed to establish a quantitative relationship between short-term and long-term treatment effects on efficacy in RA clinical trials [38]. The MBMA database was constructed using publications between 1994 and 2012, as available in the medical literature or accessible from the US FDA or EMA websites as drug labeling information. Additional data sources included conference abstracts and presentations. The constructed database included data in biologic disease-modifying anti-rheumatic drugs (DMARDs), as well as synthetic DMARDs approved or in development for RA at the time of the analysis. Only randomized controlled trials with at least 12 weeks of treatment were included in the analysis, and the clinical efficacy endpoints of American College of Rheumatology (ACR) 20, 50, and 70 responses rates (ACR20, ACR50, ACR70, respectively), as well as Disease Activity Score in 28 joints (DAS28) were included in the MBMA database. While ACR20, ACR50, and ACR70 are binary data at the patient-level, these efficacy endpoints can be summarized in terms of the proportion of patients achieving specified thresholds to be modeled in MBMA as a continuous outcome.

Using the analysis dataset developed from the MBMA database, the relationship between 3- and 6-month ACR50 treatment effects was quantified by a generalized nonlinear model, followed by a covariate assessment. The results of the MBMA show that ACR50 at 6 months is strongly correlated with that at 3 months, moderately correlated with that at 2 months, and only weakly correlated with results obtained at < 2 months. A scaling factor that reflected the ratio of 6- to 3-month treatment effects was incorporated in the model development, and the ratio was estimated to be 0.997, suggesting that the treatment effects at 3 months are approaching a “plateau”. In the covariate assessment, drug classes, baseline DAS28, and the magnitude of control arm response did not show significant impacts on the scaling factor.

The MBMA confirms the strong correlation between short-term and long-term treatment effects on efficacy in RA, indicating that the analysis results quantitatively support the use of 3-month efficacy data to predict long-term efficacy and to inform the probability of clinical success based on early efficacy readout. Consequently, a phase II POC study (GA29350) in RA patient was designed with the primary efficacy time point at 3 months [39], based in part on the results from this MBMA analysis.

#### Competitive benchmarking of fenebrutinib efficacy

GA29350 was a phase II dose-ranging study to evaluate the efficacy and safety of fenebrutinib, a Bruton’s tyrosine kinase inhibitor, in patients with moderate to severe active RA and inadequate response to previous methotrexate therapy or anti-tumor necrosis factor (anti-TNF) therapy. Three dose levels of fenebrutinib (50 mg once daily, 150 mg once daily, 200 mg twice daily) were tested in the study to obtain a broad range of exposures, with minimal exposure overlap among the dose groups, to allow a thorough investigation of the E-R relationship in patients with RA. At the end of the phase II study, a MBMA was performed to leverage publicly available information to enable direct and indirect comparisons against competitors and between study populations. The methodology and results of the analysis were published previously and are summarized here [40].

An efficacy and safety meta-analysis database were constructed in part with the existing database that was used in the previous MBMA correlating short-term and long-term treatment effects on efficacy in RA [38] and contained data published in or before 2012. The database was augmented with pertinent data published through 2017, using an additional systematic and quality-controlled procedure based on the guidelines in the Cochrane Handbook for Systematic reviews, to search for relevant published available data from the PubMed, Cochrane Library, and Embase databases using the search term “rheumatoid arthritis”. Inputs on the appropriate efficacy and safety endpoints, pre-specification of covariates to be considered for assessment, and comparators of interest were obtained from clinical science and competitive intelligence functions of the project team for database construction.

The summary data of the database were explored systematically to determine the amount of data available for each treatment, efficacy, and safety endpoint across trials and to graphically view the time-course of longitudinal endpoints, placebo effect, active treatment effect from each of the comparator treatments. Response rates of ACR20, ACR50, and ACR70 were the most prevalent efficacy endpoints in the database, and therefore, were chosen as the efficacy variables for model development using MBMA. Because of the relatively few adverse events observed in the fenebrutinib phase II trial in RA, MBMA for safety events was not conducted.

The MBMA model incorporated the effect of placebo or active treatment as a function of time, as well as the effects of drug doses, and patient population characteristics. ACR endpoints in terms of the proportion of patients achieving specified thresholds are continuous outcome variables at the summary-level, and an exponential function reflecting the increase to a maximum effect was used to describe the time-course of ACR20, ACR50, and ACR70 simultaneously. As data from different dosing schedules were available, the dose was normalized to total amount in one week in order to pool data for the same treatment across multiple trials, and the dose–response of fenebrutinib on ACR response endpoints in the MBMA model was informed by leveraging exposure-efficacy model parameters estimated using patient-level data. Model evaluation was performed using convergence diagnostics and internal validation to assess goodness of fit based on posterior predictive checks [18].

**Fig. 3:**
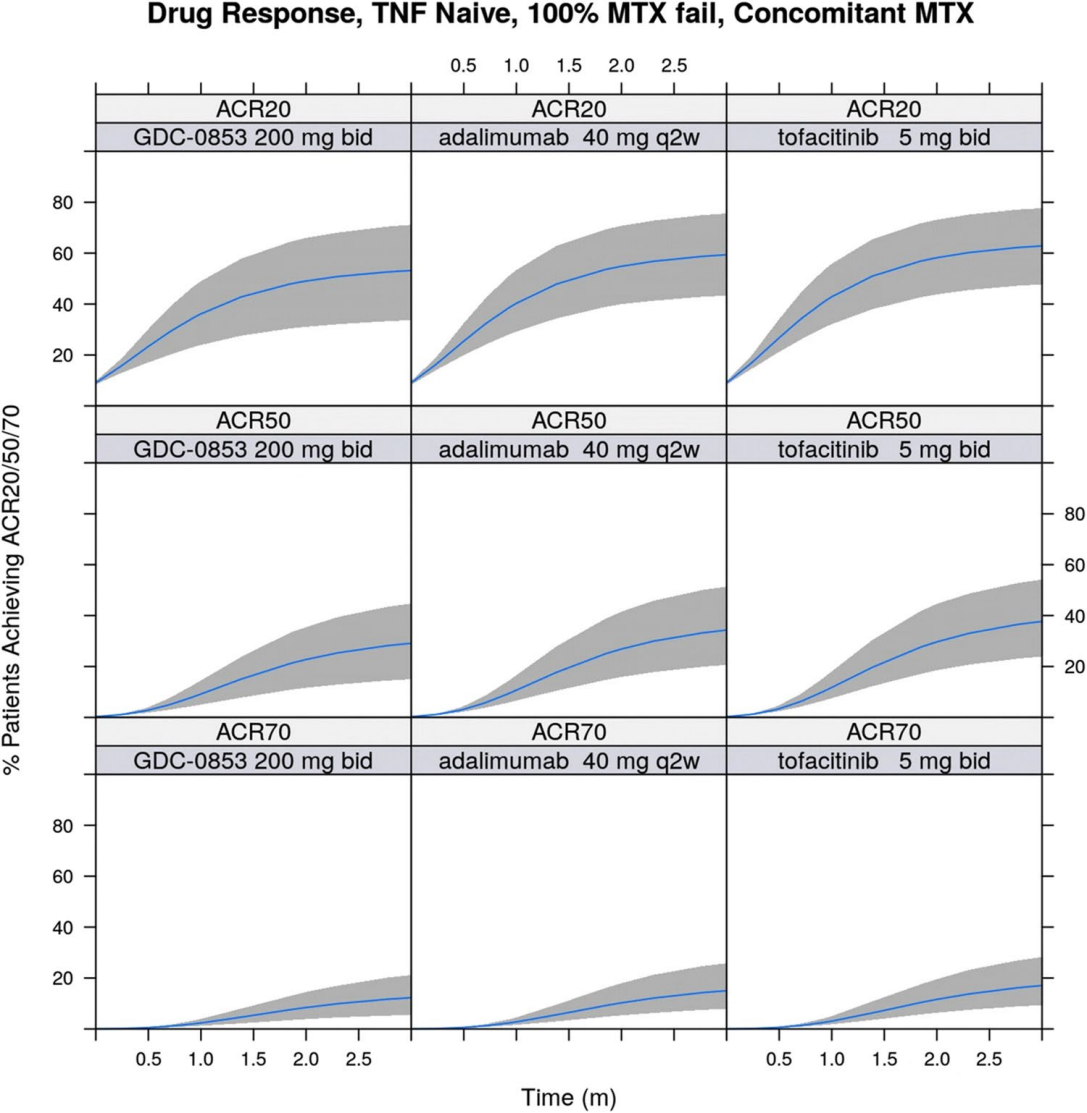
Simulations based on the rheumatoid arthritis MBMA model

The results of the MBMA showed that the ACR20, ACR50, and ACR70 response rates in the placebo and active control (adalimumab) arms of the fenebrutinib phase II trial were found to be consistent with historical data for these treatments. Covariate analysis found a high proportion (> 80%) of patients who had inadequate response to previous anti-TNF therapy, the percentage of patients who had failed previous methotrexate treatment, and concurrent methotrexate therapy had statistically significant impact on ACR response rates. Simulation based on the developed MBMA model showed that the highest fenebrutinib dose tested (200 mg twice daily) was predicted to have similar efficacy in terms of ACR20, ACR50, and ACR70 benchmarked to the registrational doses of adalimumab and tofacitinib (Fig. 3), based on direct and indirect comparison, respectively, in patients who had failed previous methotrexate treatment (but are anti-TNF naïve) at 12 weeks after initiation of treatment.

Even though the efficacy of fenebrutinib beyond 12 weeks is unknown and the correlation between short-term and long-term treatment effects on efficacy in RA may not apply to a molecule with a new MOA, such as fenebrutinib, the results from the MBMA show that fenebrutinib would not have superior efficacy when compared to multiple approved competitors of interest. Therefore, although the trial results were positive, the results from the MBMA supported the company’s decision to terminate clinical development of fenebrutinib in the RA indication, as the highest dose tested in the clinical trial would not be able to provide substantially greater benefits to patients as compared to existing therapeutic options.

## Discussion

The two case studies provided applications of MBMA in biologics (durvalumab + tremelimumab) and a small molecule (fenebrutinib) to support drug development decision-making in two different but well-studied disease areas. To increase the likelihood of success into phase III in clinical development, it is imperative to confirm that adequate target site exposure, target binding, and target engagement are established during the dosing interval before going into larger patient studies [41]. However, in the two case studies where treatment options already exist for the disease indications, the clinical development success of the investigative treatment also depends upon determining an optimal dosing regimen that provide greater efficacy benefit and/or fewer safety risks compared to previously approved therapies, potentially including other experimental molecules that are still under development for the same indication, either from the same sponsor or from the multiple pipelines of different sponsors.

Overall, the case study of fenebrutinib clinical trial in RA illustrates the positive impact such approaches can have on decision-making and clinical trial efficiency, first by allowing a shorter POC study with earlier primary efficacy endpoints to be conducted, and second by ending a project with low probability of success. The use of early endpoints enabled early planning and presumptively early initiation of phase III trials, and information on treatment/class effect, such as E-R estimates (e.g., Emax, EC50) from patient-level data, could be incorporated into the MBMA model to expedite the modeling effort, especially in treatments with novel MOAs, such as fenebrutinib. Other longitudinal MBMA models in RA have been published [15, 42, 43], and existing MBMA models can be applied for drug development in novel MOA using extrapolation, with assumption and caveats of the MBMA modeling approach presented to the project team in a transparent manner.

Although MBMA are commonly conducted for efficacy evaluations, MBMA and meta-analysis in general can assess safety and is used by regulators to evaluate drug safety [21], especially in less frequent safety outcomes, which is necessary to rely on pooled analysis [4]. In the case study with ICIs, the compiled database included reported publications from scientific journals, conference abstracts, and public presentations, and the integration of drug exposure level and potency into the MBMA framework allowed comparative landscaping from studies with different treatments to support dose justification for combination phase III study of durvalumab + tremelimumab.

### Covariate analysis

In both case studies, covariate analysis was performed as part of the MBMA model development. Summary-level data used for covariate analysis are generally not as granular or informative compared to individual-level data [15]. Therefore, covariate relationships established at the summary-level may not always be equivalent to those uncovered through patient-level mixed-effects analysis [9, 13]. Furthermore, MBMA covariates identified through statistical testing should not be interpreted solely based on a mechanistic context [44]. For example, one common prospectively explored covariates in MBMA is publication year of the citation in the database as a potential predictor of the endpoint, as the background therapy in clinical trials might change over time and could confound the treatment effect [45]. Nevertheless, the covariates found in the two case studies should be further confirmed by the individual-level data [46].

### Model evaluation

The MBMA models developed in the two case studies were evaluated using posterior predictive check, in which simulations were performed using the fitted models, and the results of the simulated data were summarized across time or drug exposure and compared to the observed data, as shown in Fig. 2B and 2C, to identify any biases and outliers. Additional methods to check for the adequacy of model fits can also include goodness-of-fit plots and summary forest plots of observed distributions vs. predicted values. Furthermore, sensitivity analyses can be conducted to assess the impact of using alternative derivation of data or a subset of data on the model development or fitting.

### Limitations of model-based meta-analysis

Certain challenges are inherent to MBMA and to meta-analyses in general, and some of the concerns are related to the source data, such as publication bias. Publication bias arises when trials with positive outcomes are more likely to be published and thus included in the meta-analysis database. To assess publication bias, funnel plots showing the relationship between the treatment effects and the standard errors of the trials included in the analysis should be examined for symmetry, and associated statistical testing, similar to Egger’s regression in the ICI case study, can be performed to detect the significance of the publication bias.

The major constraint of MBMA lies in its prominent use of publicly available summary-level data, which is not granular enough to address certain research questions that involve covariate analysis from an extensive list of potential prognostic factors. This limitation is most often mentioned by stakeholders when the researcher has access to a large database of randomized clinical trials that contain individual-level data from patients in the target population with standard-of-care treatments. Furthermore, for comparative efficacy or safety applications, relying solely on published data might not be sufficient, when the focus of stakeholders is on proprietary data that are not yet released by the competitors, in particular when the molecules from various sponsors are in similar stages of drug development. However, especially for therapeutic areas in which the individual-level data is scarce or when indirect comparison to a treatment not tested in the clinical trial, resources should be allocated to construct a MBMA database and conduct an analysis, as the insight provided by MBMA would be invaluable to support various drug development decisions.

### Additional model-based meta-analysis applications

Despite the aforementioned limitations of MBMA, besides comparative efficacy and safety as presented in the two case studies, other successful applications of MBMA have been published and a partial list with brief descriptions are shown in Table 1. Many examples in the list leveraged MBMA for multiple applications, such as the fenebrutinib case study, where a longitudinal model was developed to conduct a more comprehensive efficacy comparison.

**Table 1:** Examples of MBMA applications

Type of analysis	Applications	References
Population pharmacokinetic model development	Describe the population pharmacokinetic in a molecule with a lack of published model or to describe the pharmacokinetics in special populations	[6, 47–50, 64–67]
Longitudinal treatment effect	Characterize the time-course of efficacy endpoints (e.g., viral response) with repeated measurements per reporting unit	[12, 15, 16, 53, 68–79]
Covariate investigation	Merge data from multiple studies for the determination of covariate effects not explored in a population pharmacokinetic model	[80–85]
Dose–response (efficacy or safety) estimation	Combine data of molecules in the same class or indication to obtain an overall trend of the treatment effect	[43, 76, 86–91]
Disease progression characterization	Provide longitudinal profiles of the natural disease progression or of placebo treatment	[18, 46, 92–95]
Comparative efficacy and/or safety	Competitive benchmark and rank the treatment effects among molecules of interest	[10, 11, 31, 40, 44, 51, 85, 96–106]
Aggregation of individual data	Leverage the basic definition of MBMA, i.e., model development based on data from multiple studies	[19, 107–110]
Correlation between early and late endpoints	Allow the use of a biomarker or an early clinical efficacy time point to detect a signal of the treatment effect	[38, 42, 63, 111]
Pharmacokinetic and pharmacodynamic relationship	Establishing the relationship between exposure and an efficacy or safety biomarker, possibly a less frequently reported one and would need data from a large population to be detected	[41, 112–117]
Simulation of established MBMA models	Simulate various scenarios using established MBMA models to optimize clinical trial design	[62, 118]
Pharmacoeconomics	Incorporate cost-effectiveness into a MBMA model	[119]

An early use of MBMA involves using summary-level data available in the literature to establish population PK models for approved molecules that have published population PK models based on individual-level data (Table 1). Instead of trying to collect dense concentration–time profiles in individual patients, which are often not feasible in the post-approval setting, one potential approach that can be used to overcome this issue is to develop a population PK model using a large collection of therapeutic dose monitoring data [47]. Alternatively, MBMA can integrate individual- and summary-level PK data reported in various studies to construct a relatively complete population PK model [6, 48–50].

The placebo effect can be incorporated as "baseline" treatment effect in the model and the magnitude of the drug effect could be estimated [13]. The placebo effect over time can also be quantified separately and then be used to adjust for treatment effects, to avoid introducing bias in the estimation of relative effects of the active treatments [10, 51]. Establishing a placebo response model would be helpful for understanding the disease progression and for identifying predictors that would impact the disease progression. The placebo response model developed using MBMA could be leveraged to optimize clinical trial design, including the determination of entry criteria and maximization of relative active treatment effect [46], as well as to create a virtual comparator arm or a hybrid comparator arm, by incorporating the MBMA as a Bayesian prior, and thus reducing the number of patients needed to achieve the necessary statistical power [45]. One important consideration of developing a placebo response model using data from multiple clinical trials is when placebo-controlled trials are qualitatively different from head-to-head trials (e.g., different susceptibility to reporting bias), combining data from these two types of trials may introduce bias, if assumptions are not examined and addressed [7].

### Collaboration between pharmacometrics and other drug development functions

The two case studies illustrate the application of MBMA to support drug development decisions using a MIDD approach, and the analyses were conducted through the collaborations between a pharmacometrics group and various other functions of the project team. Based on the authors’ experience, the extent of accepting and leveraging MBMA results to support internal decisions varies greatly across project teams. It is critical that important data sources are included in the analysis database, particularly for comparative benchmarking, where an appreciation of the competitive landscape and present state of the standards of care for the disease indication of interest is critical to achieve the goals of the analysis [9]. The clinical science, competitive intelligence, and/or epidemiology functions should be consulted to obtain the most relevant and current information on the competitors of interest and to understand the placebo/disease progression. Additional collaborations are also possible with the health economics and outcomes research (HEOR) function, with which outputs from the MBMA could be integrated into other, non-clinically focused models, such as cost-effectiveness models or financial forecasting models, potentially leading to improved commercial and financial strategies [45]. Therefore, continued education and communication among project stakeholders, team collaborators, and other pharmacometric specialty experts regarding MBMA and MIDD approaches in general are warranted [1].

Another important ally of MIDD approaches is from statistics. In a survey conducted in 2017 in clinical pharmacology and pharmacometric field, the authors of the study suggested the need for greater engagement with statistical colleagues on using pharmacology principles to maximize the value of MBMA [1]. Recently a new International Society of Pharmacometrics (ISoP) Statistics and Pharmacometrics Special Interest Group sub-committee in MBMA was created, in collaboration with the American Statistical Association. The core team members of the MBMA sub-committee include those from the biotechnology/pharmaceutical industry, contract research organization, and regulatory agency, collaborating on work streams and building a MBMA community of practice within and outside of the pharmacometrics community, with major objective to initiate communication with statisticians about MBMA principles and to educate statisticians in the utility of MBMA through the identification of training opportunities [52]. Such jointly established community of practice through a professional association would further increase and solidify the collaboration and cooperation between the pharmacometric and statistical practices in the future.

### Databases and information sharing

A major impediment to conducting MBMA is associated with difficulties in constructing MBMA databases. Data sharing of summary-level data through consortia is one potential solution, but with increasing access to patient-level clinical trial data and real-world data from the clinical practice settings, the lines between patient-level and trial-level are getting increasingly blurred [9]. Most meta-analyses only include data from randomized clinical trials, but there has been a growing interest in applying to real-world data [53].

Innovative data sharing and crowdsourcing strategies [54], where a large group of people are given access to a valuable set of data to achieve a common project goal, may help unlock the full potential of meta-analyses for drug development. The crowdsourcing approach has recently been adopted by the US FDA as a pilot project to examine pooled analyses using data from pediatric clinical trials [55]. The crowdsourcing platform has also been implemented to identify a prognostic model for the prediction of survival in patients with metastatic castration-resistant prostate cancer in a crowdsourced Dialogue for Reverse Engineering Assessments and Methods (DREAM) challenge [54]. The participants of the DREAM challenge received data from the placebo and active control arms, including 150 clinical variables from the clinical trials. A major feature of this crowdsourcing effort was the removal of privacy and legal barriers associated with open access to phase III clinical trial data through a not-for-profit initiative by a consortium that broadly shares de-identified patient-level oncology clinical trial data with researchers [54]. In addition to providing a rich set of open-data that can be mined, the modeling results from such crowdsourcing platforms could advance future model development faster and further than individual research organizations working separately and would result in more efficient clinical trial design industry-wide.

### Machine learning in model-based meta-analysis

Another aspect of data science that has great potential to further expand the implementation of MBMA in drug development is machine learning (ML). As modeling, simulation, and ML are often used for the same purpose, these three approaches could join forces to exploit their relative merits [56]. One such realization involves the automation of many steps of the systematic review process, including search, screening, and data extraction [57, 58], which can greatly expedite the MBMA database-building process. Initial screening through abstract reviews is typically the most labor-intensive and time-consuming step of conducting a meta-analysis because the traditional systematic review process is rigorous but not efficient. Conducting a single systematic review requires more than 1000 h of highly skilled labor [57], but is associated with a low yield rate of approximately 2.94% [59]. A particular subfield of ML called natural language processing (NLP) can significantly reduce the manual workload of the systematic review process by performing text mining and classification of the publication abstracts and even data extraction [57]. ML methods can also be leveraged to estimate the probability of whether an abstract should be included, which can alleviate the amount of manual labor required to construct a MBMA database [57]. However, even with the implementation of the currently available ML approaches, the MBMA database-building process would be semi-automatic at best, and the NLP-based procedures should still be followed by human review of identified studies and extracted data.

A major feature of ML is its ability to process large-scale data, and sophisticated methodologies have been developed in the ML field to cope with data management and modeling issues. For example, ML-based statistical methods can improve the convergence of the regression methods utilized in the development of predictive models [60], including those used for MBMA. Also, ML-based methods have been developed for the imputation of missing features as a function of all other features and could be leveraged in MBMA as well. Another potential future use of ML for missing features in MBMA databases is the implementation of deep learning algorithms, which can learn features from the data without assumptions and may outperform previous approaches in imputation tasks [61].

## Conclusion

**Fig. 4:**
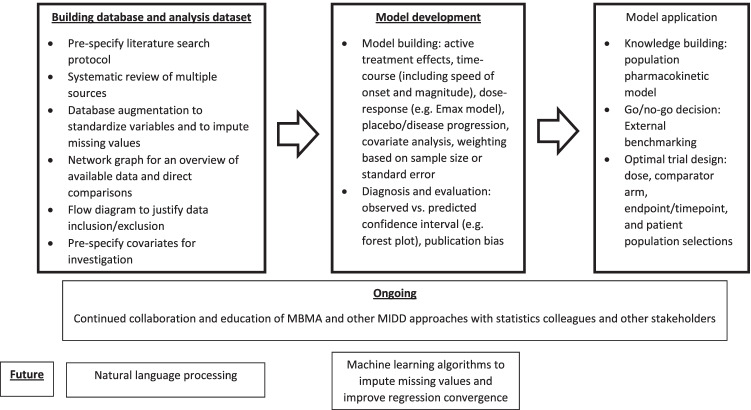
Schematic of overall MBMA process

There are various aspects of the MBMA database-building and model development steps, as shown in Fig. 4, that will improve as the field continues to expand. In the past, publication bias had been a genuine concern in conducting meta-analysis, when clinical trials with positive results were more likely to be published than those with negative or inconclusive results. However, after the US FDA Amendment Act (FDAAA) mandated clinical trial registration and reporting of results on ClinicalTrials.gov in 2007, some of the publication bias concerns have been reduced [24].

In general, information provided by a MBMA can be used to: (i) compare new compounds to hypothetical controls comprised of either emerging or historical, existing treatment options, (ii) link short-term (e.g. biomarker) responses to long-term clinical efficacy and safety end points, (iii) integrate internal and external data to better characterize dose–response and optimize dose selection and, (iv) identify or bridge a target population for trial design [24, 62]. By summarizing the overall evidence from multiple studies, MBMA is an integrated component of the MIDD toolbox. MBMA results often inform molecule-level “go/no-go” decisions but also can shape portfolio-level strategy when comparing multiple internal assets developed for a common indication [63]. MBMA is a holistic approach that examines the totality of available evidence and allows better informed decision-making to speed up development of a drug candidate with improved benefits for patients, consistent with model-based strategies in PDUFA VI to support drug development.
